# What should we be selecting for? A systematic approach for determining which personal characteristics to assess for during admissions

**DOI:** 10.1186/1472-6920-12-105

**Published:** 2012-11-05

**Authors:** Peter Conlon, Kent Hecker, Susan Sabatini

**Affiliations:** 1 Dean’s Office, Ontario Veterinary College, University of Guelph, Guelph, ON, Canada; 2 Department of Veterinary Clinical and Diagnostic Science, Faculty of Veterinary Medicine, Department of Community Health Sciences, Faculty of Medicine, Medical Education Research Unit, University of Calgary, Calgary, AB, Canada; 3 G380 Health Sciences Centre, 3330 Hospital Drive NW, Calgary, AB, T2N 4N1, Canada

**Keywords:** Personal characteristics, Admissions, Selection criteria, Survey

## Abstract

**Background:**

Admission committees are responsible for creating fair, defensible, reliable, and valid processes that assess those attributes considered important for professional success. There is evidence for the continuing use of academic ability as a selection criterion for health professional schools; however, there is little evidence for the reliability and validity of measures currently in place to assess personal characteristics. The Ontario Veterinary College (OVC) initiated a review of its admissions criteria in order to implement an evidence-based method to determine which characteristics veterinary stakeholders consider important to assess for admission.

**Methods:**

Eleven characteristics were identified by the OVC Admissions Committee and a survey was sent to all licensed veterinarians in Ontario (n=4,068), OVC students (n=450), and OVC faculty, interns and residents (n=192). A paired comparison method was used to identify the relative rank order of the characteristics, and multivariate analysis of variance with post hoc analyses was used to determine between group differences in the returned survey data.

**Results:**

Surveys were returned from 1,312 participants (27.86% response rate; female 59.70%). The relative rank of the characteristics was reasonably consistent among participant groups, with ethical behaviour, sound judgment, communication, and critical and creative thinking being ranked as the top four. However, the importance of certain characteristics like communication and empathy were perceived differently by groups. For instance, females scored communication (*F*(1, 1289) = 20.24, *p* < .001, *d* = .26) and empathy (*F*(1, 1289) = 55.41, *p* < .001, *d* = 0.42) significantly higher than males, while males scored knowledge of profession (*F*(1, 1289) = 12.81, *p* < .001, *d* = 0.20), leadership (*F*(1, 1289) = 10.28, *p* = .001, *d* = 0.18), and sound judgment (*F*(1, 1289) = 13.56, *p* < .001, *d* = 0.21) significantly higher than females.

**Conclusions:**

The data from the paired comparison method provide convergent evidence for the characteristics participant groups identify as most important in determining who should be admitted to a veterinary program. The between group analyses provides important information regarding characteristics most important to various subgroups; this has implications for what characteristics are selected for at admission as well as on who is selecting for them.

## Background

Personal characteristics other than academic ability have long been recognized as requirements for economic and career success in veterinary medicine in North America. While these characteristics or attributes have been increasingly emphasized within the Doctor of Veterinary Medicine (DVM) curriculum (i.e. teaching communication skills or providing ethical dilemmas for students to work through), they require years of development [1]. Presuming that a student will acquire these characteristics while also dealing with the DVM program course work is probably incorrect. Given the limitations of adding to an already overwhelming curriculum, a more realistic goal is to enhance personal characteristics already in place. Selection of students into the DVM program should be based on their academic excellence and possession of the attributes deemed most necessary for success in the DVM program and in the veterinary profession.

Before discussing how best to assess personal characteristics at the time of admission, it is important to identify which personal characteristics various stakeholders believe are important to assess at the time of admission. The Pew Report, released in 1988, evaluated the veterinary profession and discussed changes essential to ensure that it has a viable future. The necessary characteristics for veterinarians proposed in the Pew report include: communication skills; general understanding of the world; sensitivities to cultures and people; scientific and professional behavior; desire for sustained scholarship; commitment to the betterment of humanity; personal management skills; compassion for animals and people; and personal integrity and ethics [2]. More recent studies have also identified the importance of personal characteristics for veterinarians. The KPMG LLP report stated that “While there is ample evidence that the scientific and clinical skills of the profession remain very high, there is also evidence that veterinarians lack some of the skills and aptitudes that result in economic success. Additionally, there is evidence that veterinarians’ self-perception of their abilities and their perception of what they can contribute to society potentially limit the professional and economic growth of the veterinary medical profession” (pg. 162) [3]. A study conducted by Ilgen et al. suggests that current admissions procedures in North American veterinary colleges may be screening out applicants with desirable attributes related to non-technical competencies [4].

The idea that personal attributes are necessary for the economic and career success of veterinarians has been recognized within the veterinary profession in North America. The Canadian Veterinary Medical Association, in its National Action Plan developed from the Task Force Report on the Future of the Veterinary Profession, made the following recommendation concerning the selection of students by the veterinary colleges in Canada: “To ensure that individuals with good interpersonal skills and broad interests, not only individuals with exceedingly high academic qualifications, gain entry into veterinary medicine, the Task Force recommends that veterinary colleges: adopt a basic academic standard for admission, beyond which candidates are assessed on a broad range of aptitudes as proposed in the PEW report; and recognize aptitude testing and personality profiling as pivotal in the selection process” (pg.408) [5]. Lewis and Klausner stated that the core competency components that must be taken into account when selecting people who will be successful in their careers include: technical [cognitive] competencies that comprise the knowledge, skills, and experiences that lead to success in a particular job that can be gained in a short period; and non-technical [non-cognitive] competencies that consist of personality traits, abilities and core interests, values, and motivations which are developed over years and are less amenable to training and planned change [6].

This implies that academic ability and aptitude for veterinary medicine are necessary but not sufficient for success in the profession. In response to these findings, a consortium of veterinary colleges was created to help identify problems in the profession that may be leading to decreasing career success and satisfaction. Personnel Decisions International (PDI) was hired by the consortium to identify the non-technical competencies related to success in the veterinary profession and to determine how these competencies varied in importance for success in various veterinary career paths [6]. While some of the competencies were found to be specific to certain career paths, overall, they were found to be consistent across a range of veterinary career settings. This study was the first to systematically assess characteristics other than academic ability within the veterinary profession. Lists of characteristics defined as necessary for success as a veterinarian have been previously generated in the literature; however, the non-cognitive competencies developed by Lewis and Klausner were defined in behavioural terms, which they recommended should be assessed during admissions and veterinary school [6]. In medicine, Albanese et al. identified 87 personal characteristics important for medicine, however, not all of these could be translated into qualities that can be measured at the time of admission [7]. Hecker et al. reviewed attributes that veterinary schools reported as being measured at the time of admission. Thirty-seven attributes were reported with each school reporting up to 6 attributes being assessed at the time of admission [8].

The Veterinary Medical College Application Services (VMCAS) website includes “college descriptor pages” that list the selection process for each of the veterinary schools that are members of the Association of American Veterinary Medical Colleges (AAVMC) [9].

In reviewing the 28 schools in the USA and 5 in Canada, all veterinary colleges list grade point average (GPA) requirements. All but 3 require a supplemental/standardized test be it the Graduate Record Examination (GRE) or Medical College Admission Test (MCAT). Non-academic attributes are assessed in various ways and may include references, veterinary and/or animal experience, extracurricular activities, volunteer and work experiences, personal statements/essays and interviews. Five of the colleges did not require an interview as part of their admissions process. Of the colleges that reported the interview style used, the University of Calgary (UCVM), the University of California – Davis, Virginia Maryland, and the Ontario Veterinary College (OVC) use the Multiple Mini Interview (MMI) [9]. The MMI is a series of timed interview stations, typically 8 – 10, and each station is 10 minutes in length. The candidate is presented with a scenario or task (they have 2 minutes to read the scenario), they are then asked to discuss the scenario with the interviewer or perform the respective task. The interviewer then rates candidate performance on certain attributes, i.e. communication skills. The weighting formula used to determine an applicant’s ranking differed for each college.

Veterinary schools, however, rarely report how the lists of personal characteristics applicants are required to possess are determined. At a minimum, characteristics are selected by schools’ admissions committees that are reported to be a reflection of the goals and objectives of the respective school. Recently there have been reports of empirical methods used for the selection of admissions criteria. Reiter and Eva proposed the use of the paired comparison method to create a rank order list of characteristics considered important for medical students to possess [10]. Using this method, 292 community members, faculty members and medical students were surveyed and asked to compare seven characteristics. They reported that subgroups rank ordered the attributes in a consistent order with “ethical” being the most important characteristic of the seven. Lambe and Bristow used a Delphi method to determine whether they could identify common attributes considered important for the medical profession [11]. Ten participants were asked to consider 20 attributes and rate them on a 1 to 5 scale (not at all important to always important) and to rank order the 20. The top three attributes identified were, “recognition that patient care is the primary concern of the doctor”, “probity (being honest, trustworthy and acting with integrity)”, and “good communication and listening skills.” It is beyond the scope of this paper to review selection methods used for admissions to assess academic ability and personal characteristics. If interested please refer to Prideuax et. al.s recent consensus document regarding selection measures used at admissions [12].

While identification and rank ordering of attributes is important, what needs to be explored is whether certain attributes are considered as being more important among different demographic groups. For example do males perceive communication skills as more important than females? Is there a difference amongst age categories? Is there a difference between practitioner types? Is there a difference amongst education level? If there are differences, these could have an impact on admissions decisions and might have to be taken into account during the admissions process. Therefore, the purpose of this study was twofold. First, we wanted to systematically determine which personal characteristics members of the veterinary profession in Ontario deemed most important for making admission decisions to the Ontario Veterinary College. Second, we wanted to explore whether there were between group differences in how members of the profession scored attributes.

## Methods

### Survey development

The OVC admissions committee regularly reviews the process by which applicants are assessed for admission to the DVM Program. Students applying to OVC from 1996 to 2008 were reviewed based upon their GPA and a biographical submission. Some students in each admission cohort were interviewed (traditional, panel style interview format) and others were not. This decision was based on a flagging system where positive and negative flags were assigned to academic ranking, background information, and referee assessments. In 2000, the MCAT requirement was introduced. The OVC admissions committee decided to revise the DVM admissions process again beginning in 2008. Applicants continue to be assessed on GPA, MCAT scores and biographical submission. The Multiple Mini Interview (MMI) was adopted in 2010 to assess for personal characteristics deemed important for veterinary students. The committee wanted a systematic method of identifying the most important attributes to be assessed during the MMI. These characteristics were identified based upon the goals and objectives of OVC and published data in the veterinary and health professional literature and are listed below [3,6,10,13,14].

Altruism- Unselfish regard for, or devotion to, the welfare of others.

**Communication**- Communicates effectively by oral, written and electronic means, and listens respectfully.

**Collaboration**- Demonstrates the ability to work cooperatively and effectively with others.

**Critical and Creative Thinking**- Able to reason logically; considers multiple viewpoints and critically appraises information. Seeks new ideas to create new approaches to problems/challenges.

**Empathy**- Understands another’s feelings and perspective and the ability to communicate one’s empathetic feelings and understandings to another by verbal and/or non-verbal means.

**Ethical**- Demonstrates honesty, integrity and respect, and makes principled decisions.

**Knowledge of the Veterinary Profession**- Appreciates the multiple career paths within the veterinary profession. Evidence of experience in one or more areas of the veterinary profession. Basic understanding of the self-regulation of the profession. Demonstrates advanced thought/knowledge of the issues facing the profession.

**Leadership/Mentoring**- Motivates others to achieve their goals - Builds knowledge and skills in others, and gives timely and accurate feedback.

**Personal Management Skills**- Makes decisions independently when warranted and is capable of working alone when necessary - Demonstrates adaptability and resilience by using skills and experiences to handle a wide variety of challenges and opportunities. Demonstrates organization and discipline in order to reach goals.

**Self–initiated Learning**- Demonstrates a commitment to on-going learning and self-evaluation. Broadens perspective by pursuing interests outside the profession.

**Sound Judgment**- Makes decisions on the basis of logic, evidence, experience and accepted practice.

When the current curriculum at OVC was established, the “DVM 2000” Steering Committee defined the knowledge, skills and behaviours expected of an entry-level veterinarian and established them as behavioural objectives for the DVM curriculum in a document called the “Professional Competencies of Canadian Veterinarians” [14]. The non-academic attributes listed in this document, along with the attributes found in the Pew Report and the Lewis and Klausner paper [3,6] were compared and found to have overlap and similarities. Another comparison was made to the list of non-academic characteristics defined by Reiter and Eva as being important for physicians to have as the professions are similar in many regards [10]. As a result of these comparisons and utilizing the framework proposed by Reiter and Eva [10], a list of 11 non-academic characteristics and their definitions were established by the admissions committee.

This study received ethics clearance through the University of Guelph Research Ethics Board. A survey was created listing 55 pairs of the 11 characteristics in a randomized order (e.g. communication -OR- ethical). Participants were asked to choose one characteristic from each pair. Demographic information was also requested including: sex, age, current employment status (student, practitioner, faculty member etc.), clinical practice type (if applicable), and practice owner vs. non-owner status (if applicable). Members of the Ontario Veterinary Medical Association (OVMA) Board of Directors (n=34) were asked to pilot the survey as this board represents a broad cross section of the veterinary profession in Ontario. 13 responses (38%) were received and revisions to the survey were made based on feedback from the participants (an excerpt of the survey is provided in Table 1).

**Table 1:** Example of the paired comparison survey instrument*

**For each pair of characteristics outlined below, please circle the characteristic that you consider more important in determining who should be admitted to the DVM Program at OVC. You must choose one characteristic from each pair or your responses cannot be analyzed.**	
**1.**	Altruism **-OR-** Knowledge of the Veterinary Profession
**2.**	Critical and Creative Thinking -OR- Knowledge of the Veterinary Profession
**3.**	Collaboration **-OR-** Empathy
**4.**	Collaboration **-OR-** Personal Management Skills
**5.**	Sound Judgment **-OR-** Self–initiated Learning
**6.**	Altruism **-OR**- Empathy
**7.**	Critical and Creative Thinking -OR- Communication
**8.**	Knowledge of the Veterinary Profession **-OR-** Self–initiated Learning
**9.**	Sound Judgment **-OR-** Altruism
**10.**	Critical and Creative Thinking **-OR-** Altruism
**11.**	Self–initiated Learning **-OR-** Personal Management Skills
**12.**	Sound Judgment **-OR-** Communication
**13.**	Critical and Creative Thinking **-OR-** Personal Management Skills
**14.**	Knowledge of the Veterinary Profession -OR- Collaboration
**15.**	Collaboration **-OR-** Sound Judgment
**16.**	Ethical -OR- Empathy
**17.**	Personal Management Skills **-OR-** Sound Judgment
**18.**	Sound Judgment **-OR-** Critical and Creative Thinking
**19.**	Ethical **-OR-** Self–initiated Learning
**20.**	Ethical **-OR-** Personal Management Skills
**21.**	Collaboration -OR- Critical and Creative Thinking
**22.**	Collaboration **-OR-** Leadership/Mentoring
**23.**	Personal Management Skills **-OR-** Communication
**24.**	Altruism **-OR-** Collaboration
**25.**	Personal Management Skills **-OR-** Altruism

### Participants

The final version of the survey and a cover letter were sent to all stakeholder groups: all licensed veterinarians in Ontario (n=4,068), OVC students (n=450), and OVC faculty, interns and residents (n=192). Licensed veterinarians are those holding a license issued by the College of Veterinarians of Ontario, the regulatory body for veterinary medicine in the province. Students are defined as being enrolled in the Doctor of Veterinary Medicine degree program. Interns hold the DVM degree and are engaged in one year of post-graduate clinical training. OVC does not employ private practitioners to work in its teaching hospital. Participants were given three choices as to how to respond – by mail, fax or on-line. The survey took 15–20 minutes to complete.

### Statistical analyses

The paired comparison method was used to rank order the attributes considered most important by respondents [15]. For each pair (e.g. Altruism -or- Knowledge of the Veterinary Profession) a score was calculated for each characteristic. For instance 710 respondents selected altruism and 599 selected knowledge of the veterinary profession. This was converted into a raw proportion (0.54 for altruism, 0.46 for knowledge of the veterinary profession). These values were then used to create an 11 by 11 matrix where the columns represented the score for the attribute. For example, in the column for altruism the score compared to knowledge of the veterinary profession was 0.54, and the score for knowledge of veterinary medicine (column), when compared to altruism (row) was 0.46. The same attributes compared to one another (altruism vs. altruism) was 0.50. These proportions were then converted into a Z score, and an average Z score (+3) was calculated for each attribute. This average Z score was then used to assign a rank (see Reiter and Eva and Streiner and Norman for worked examples of this analysis) [10,16]. Rank orders were created for the entire data set and responses grouped according to sex, age, education level, clinical practice type and owner vs. non-owner status.

Multivariate analysis of variance (MANOVA) was used to determine if there were between group differences (i.e. between males and females) on the scores of various attributes (for instance communication, ethical and sound judgment). Scores from the pair wise comparison (0 or 1) for each attribute were added together to create an attribute score for each characteristic for each individual. The maximum score that could be achieved for each characteristic was 10. MANOVA was used because this method has more power to detect group differences than multiple ANOVAs and can account for the inter-correlations between the responses [17]. Post hoc tests (Tukey’s HSD) and univariate analyses were subsequently run to identify the between group differences. The effect size were estimated using Cohen’s *d* (*d* ) and was interpreted using Cohen’s classification of small (.20), medium (.50) and large (.80) [18].

Response bias was assessed by comparing sex, group membership, and employment type demographic information from the respondents (in percentages) to provincial demographic data collected by the College of Veterinarians of Ontario (CVO) [19]. To calculate bias, the following formula was used: (survey data group percentage) – (CVO data group percentage). Values of 0 indicate perfect agreement between respondents and the CVO, positive values indicate the trait to be emphasized within the respondent data, negative values show the trait to be under emphasized in the respondent data.

## Results

Completed surveys for the paired comparison responses were returned from 1,312 participants (27.86% response rate; female 59.70%). Of these 995 were practitioners (which includes 35 industry veterinarians, and 47 government veterinarians; response rate of 24.50%), 78 were faculty members (40.63% response rate) and 133 were students (29.56% response rate). One hundred-six respondents did not self-identify an employment group. For area of clinical practice, 715 identified small animal, 155 identified mixed animal, 42 identified food animal and 40 indicated equine. Four hundred sixty-five self-identified as practice owners while 468 identified themselves as associates or locums. For age categories, there were 259 20–30 year-olds, 306 31–40 year-olds, 310 41–50 year-olds, 263 51–60 year-olds and 174 > 61 year-olds.

Typically, response rates to surveys targeting veterinarians in Ontario range from 20-50% depending on the type of survey being conducted [20-22]. The greatest response rate difference was by sex at 7.40% (female were more likely to respond than males). In all other categories, there was between a 2.39% and 4.09% response rate difference. While the response rate was low (27.86%), the bias was less that 8% indicating the respondent sample obtained is quite similar to the target population.

### Paired comparison analyses

Overall mean weighted z-scores are presented in Table 2. From the 1,312 responders, the top four characteristics were ethical behaviour, sound judgment, communication, and critical and creative thinking. The top four were focused on as we felt they align with the goals and objectives of our school and we could create interview scenarios that could encompass those attributes. We were then interested to determine if there were any differences in the rank order and relative values between sex, age, self-identified practitioner type, practitioner owner vs. non owner and employment status.

**Table 2:** **Weighted *z *–scores for all participants and male vs. female respondents**

**Attribute**	**Overall mean score**		
	(n = 1312)*	**Males (n = 519)**	**Females (n = 772)**
Ethical	3.74	3.73	3.75
Sound Judgment	3.54	3.62	3.50
Communication	3.53	3.43	3.60
Critical and Creative Thinking	3.45	3.45	3.45
Personal Management Skills	3.02	3.05	3.0
Empathy	3.01	2.83	3.04
Collaboration	2.74	2.83	2.87
Self-Initiated Learning	2.71	2.75	2.60
Leadership/Mentoring	2.48	2.56	2.43
Altruism	2.47	2.34	2.36
Knowledge of the Veterinary Profession	2.32	2.41	2.24

* overall mean Z-score +3.

The top 4 attributes identified above were consistently ranked between these various categories with some fluctuations between groups (Tables 2, 3, 4, 5, 6). The z scores were significant correlated for sex (*r* = .97, p< .001), for self-identified practitioner type (r’s between .92 and .99, p<.001), for employment status (*r*’s between .86 and .96, p <.001), for owner versus non-owner (*r* = .98, p< .001) and for age category (*r*’s between .95 and .99, p< .001).

**Table 3:** **Weighted *z *–scores for all participants and for each practitioner type**

**Attribute**	**Overall mean score**	**Small animal (n = 715)**	**Food Animal (n = 42)**	**Equine (n = 40)**	**Mixed Animal (n = 155)**
Ethical	3.74	3.75	3.76	3.59	3.77
Sound Judgment	3.54	3.54	3.65	3.82	3.54
Communication	3.53	3.61	3.42	3.31	3.46
Critical and Creative Thinking	3.45	3.48	3.61	3.78	3.44
Personal Management Skills	3.02	3	3.11	3.03	3.05
Empathy	3.01	3.07	2.57	2.77	2.94
Collaboration	2.74	2.84	2.98	2.89	2.85
Self-Initiated Learning	2.71	2.72	2.92	2.71	2.76
Leadership/Mentoring	2.48	2.43	2.67	2.37	2.52
Altruism	2.47	2.31	2.24	2.34	2.39
Knowledge of the Veterinary Profession	2.32	2.25	2.06	2.39	2.27

**Table 4:** **Weighted *z *–scores for all participants and for student and employment status**

**Attribute**	**Overall mean score**	**DVM students (n=133)**	**Practitioner (n=913)**	**Faculty member (n= 78)**	**Industry veterinarian (n = 35)**	**Government veterinarian (n = 47)**
Ethical	3.74	3.58	3.75	3.84	3.81	3.93
Sound Judgment	3.54	3.48	3.56	3.61	3.59	3.54
Communication	3.53	3.61	3.58	3.28	3.59	3.42
Critical and Creative Thinking	3.45	3.12	3.50	3.62	3.40	3.43
Personal Management Skills	3.02	3.05	3.07	2.85	2.90	3.02
Empathy	3.01	2.93	3.08	2.76	2.71	2.96
Collaboration	2.74	3.00	2.92	2.80	2.80	2.74
Self-Initiated Learning	2.71	2.74	2.80	2.96	2.77	2.63
Leadership/Mentoring	2.48	2.49	2.56	2.62	2.82	2.46
Altruism	2.47	2.59	2.42	2.32	2.14	2.45
Knowledge of the Veterinary Profession	2.32	2.41	2.40	2.34	2.46	2.41

**Table 5:** **Weighted *z *–scores for all participants and for practice owners and non-owners**

**Attribute**	**Overall mean score**	**Owner (n= 465)**	**Non-owner (n=468)**
Ethical	3.74	3.74	3.74
Sound Judgment	3.54	3.63	3.46
Communication	3.53	3.46	3.67
Critical and Creative Thinking	3.45	3.47	3.51
Personal Management Skills	3.02	3.10	2.95
Empathy	3.01	2.96	3.08
Collaboration	2.74	2.82	2.88
Self-Initiated Learning	2.71	2.74	2.73
Leadership/Mentoring	2.48	2.45	2.46
Altruism	2.47	2.32	2.31
Knowledge of the Veterinary Profession	2.32	2.30	2.21

**Table 6:** **Weighted *z *–scores for all participants and age groups**

Attribute*	**Overall mean score**	20-30* (n = 259)	31-40* (n =306)	41-50* (n = 310)	51-60* (n = 263)	>60* (n =174)
Ethical	3.74	3.62	3.68	3.79	3.92	3.71
Sound Judgment	3.54	3.45	3.53	3.59	3.57	3.58
Communication	3.53	3.64	3.66	3.58	3.37	3.32
Critical and Creative Thinking	3.45	3.32	3.53	3.50	3.48	3.41
Personal Management Skills	3.02	3.00	3.01	3.07	3.02	2.99
Empathy	3.01	3.00	3.05	2.97	2.95	2.91
Collaboration	2.74	2.97	2.83	2.78	2.82	2.89
Self-Initiated Learning	2.71	2.71	2.73	2.73	2.73	2.81
Leadership/Mentoring	2.48	2.49	2.42	2.44	2.54	2.58
Altruism	2.47	2.46	2.29	2.27	2.40	2.36
Knowledge of the Veterinary Profession	2.32	2.33	2.27	2.29	2.23	2.46

*years of age.

### Do constituent groups place the same importance on each attribute?

While the paired comparison method provides important information regarding the relative values and the rank order of the attributes relative to one another, we were also interested in whether there were between group differences in the weightings of specific attributes. Effect sizes and significant differences in paired personal characteristics were calculated between respective groups.

#### Is there a difference between sexes?

Males and females differed in which characteristics they perceived to be most important. (Wilk’s Lambda = .93, *F*(11,1279) = 8.45, *p* < .001). Females scored communication (*F*(1, 1289) = 20.24, *p* < .001, *d* = .26) and empathy (*F*(1, 1289) = 55.41, *p* < .001, *d* = 0.42) significantly higher than males, while males scored knowledge of profession (*F*(1, 1289) = 12.81, *p* < .001, *d* = 0.20), leadership/mentoring (*F*(1, 1289) = 10.28, *p* = .001, *d* = 0.18), and sound judgment (*F*(1, 1289) = 13.56, *p* < .001, *d* = 0.21) significantly higher than females.

#### Is there a difference between practice owner and non-owner?

There were significant differences in scores between owners and non-owners (Wilk’s Lambda = .93, *F*(11, 921) = 5.40, *p* < .001). The non-owners scored communication skills (*F*(1,931) =24.39, *p* < .001, *d* = 0.32) and empathy (*F* (1,931) =9.09, *p* = .003, *d* =0.20) higher than the owners. Practice owners scored personal management skills (*F* (1,931) =15.18, *p* < .001 *d* = 0.26) and sound judgment (*F* (1,931) =17.78, *p* < .001, *d* =0.28) higher than the non-owners.

#### Are there differences between practitioner types?

There was a significant difference between practice type (Wilk’s Lambda = .91, *F*(33, 2764.23)= 2.70, *p* < .001). Those in small animal practice rated communication skills higher than those practicing in equine (Tukey’s HSD, *p* < .04 *d* = 0.49) and mixed animal (*p* < .04 *d* = 0.24) practices. Equine practitioners rated critical and creative thinking significantly higher than mixed animal practitioners (*p =* .03, *d* = 0.49). Small animal practitioners rated empathy significantly higher than food animal (p < .01, *d* = 0.83) and equine practitioners (*p* < .01, *d* = 0.51). Finally, equine practitioners rated sound judgment significantly higher than did small animal practitioners (*p =* .04, *d* = 0.43).

#### Are there differences between student and employment statuses?

There were differences in how attributes were scored by student/employment status (Wilk’s Lambda = .87 *F*(44, 4558.42) = 2.91, *p* < .001). DVM students rated altruism significantly higher than did practitioners (Tukey’s HSD, *p =* .001, *d*= 0.36), DVM students and practitioners rated communication skills significantly higher than did faculty members (both *p*s =.001, *d*’s = 0.58 and 0.45 respectively), and DVM students rated collaboration higher than did practitioners, faculty members and government veterinarians (all *p*s < .05, *d*’s = 0.31, 0.42, and 0.52 respectively). Faculty members, government veterinarians and practitioners rated critical thinking skills significantly higher than DVM students (all *p*s <.05, *d*’s = 0.78, 0.47, and 0.62 respectively). Practitioners rated empathy significantly higher than faculty members (p = .003, *d* = 0.43). Veterinarians in industry rated leadership/mentoring skills significantly higher than practitioners and DVM students (both *p*s < .05, *d*’s = 0.58 and 0.51 respectively). Finally, faculty members scored self-initiated learning significantly higher than did DVM students, practitioners and government veterinarian (all *p*s < .05, *d*’s = 0.39, 0.45, and 0.57 respectively).

#### Are there differences among age groups?

There were differences in how attributes were scored by respondents in the various age categories (Wilk’s Lambda = .89 *F*(44, 4963.95) = 3.58, p < .001). Post hoc tests (Tukey HSD) revealed that 20–30 year-old respondents, 31–40 year-old respondents, and 41–50 year-old respondents scored communication skills significantly higher than both 51–60 (all *p*s < .001, *d*’s= 0.46, 0.46, 0.34 respectively) and >61 year-olds (all *p*s < .001, *d*’s = 0.55, 0.54, 0.44 respectively) . The 20–30 year-old respondents scored collaboration skills significantly higher than all the other age categories except the > 61 year-olds (all *p*s < .01, *d*’s = .28, 0.38, 0.29). The 31–40 and 41–50 year-olds scored critical and creative thinking skills significantly higher than the 20–30 year-olds (both *p*s < .02, *d*’s = 0.32, 0.26). The 51–60 year-olds scored ethical behaviour significantly higher than both the 20–30 and 31–40 year olds (all *p*s < .01, *d*’s = 0.39, 0.31). The >61 year-old category scored knowledge of the veterinary profession higher than the 51–60 year-old group (*p*= .03, *d* = 0.29).

## Discussion

The results of this study are: 1) the overall mean weighted z scores resulted in a relatively consistent rank order with the top four characteristics being ethical behaviour, communication, sound judgment, and critical and creative thinking; and 2) while there were significant correlations between the weighted z scores between respective groups, MANOVA results showed significant differences in the importance that the respective groups place on the attributes. The majority of the effect sizes were small with some being moderate and one large.

There is strong support for the argument that health professional students should be selected based upon their academic ability and personal characteristics. While the methods used to assess academic ability are reliable and valid [23] there is scant evidence of reliability and validity of the selection measures used for personal characteristics, or even which method can assess for which personal attributes. The purpose of this study was to identify the personal characteristics we wanted to assess through our interview process, the MMI. As an interview process the MMI has been shown to be reliable for veterinary admissions [24] and there are reports of its validity [25]. Currently, the MMI at OVC consists of eight 10 minute stations. The scenarios were created to assess ethical behavior [2], sound judgment [2], critical and creative thinking [2], empathy [1] and knowledge of the veterinary profession [1] within a veterinary related context [8].

Communication skills are assessed at each station with this attribute grade being weighted equally to the other attributes in the final MMI scoring method. While knowledge of the veterinary profession ranked lowest in our survey, it is the expectation of interviewers and applicants to discuss this attribute and so one station is dedicated to this. Personal management skills and empathy scores differ by 0.01 (3.02 and 3.01 respectively). As creating scenarios to assess empathy are less onerous, this attribute was chosen over the slightly higher ranked personal management skills. Attributes are assessed at least once in the circuit and up to 2–3 times.

For OVC, this data driven, empirical approach provides evidence for the personal characteristics considered necessary for success in veterinary school and veterinary practice by the various constituent groups. The consistency in the rank order among the various groups indicates that, given the attributes proposed, the same characteristics are considered important to select for at the time of admissions. The four attributes, ethical behavior, communication, sound judgment and critical and creative thinking are somewhat consistent with characteristics identified in similar types of studies. For instance, in medicine, Reiter and Eva reported the top characteristics as ethical and communication [10]. The PDI consortium, while not specific to admissions, reported their most important characteristics as agreeableness, self-esteem and conscientiousness [6].

While the rank order are similar across groups, differences in how participant groups view the importance of various personal characteristics may have an impact on applicants’ admission scores, and could affect performance within school and relationships once in practice. Female respondents placed more importance on communication and empathy than did their male colleagues, while male respondents placed more importance on sound judgment, leadership/mentoring and knowledge of the veterinary profession. We must caution not to over interpret these findings as the effect sizes were small (range from .18 to .42) [26]. Younger, non-practice owners with a small animal background typically rated communication and empathy higher than other groups in each category while critical and creative thinking was considered increasingly important with age, as was ethical behavior and knowledge of the veterinary profession. These differences among student and non-student groups may affect how students interact with faculty during their time at OVC and after graduation with graduate veterinarians when they are interviewing for positions and subsequently during employment. There may also be intergenerational effects on school and employment since those under 50 put more emphasis on the importance of communication skills. Employment type may shape attitudes regarding which attributes are deemed most important for success in the veterinary profession, or inherent beliefs regarding these attributes may influence which career path a veterinarian chooses to follow.

Differences in how participant groups value personal characteristics may have significant consequences on the results of the admissions process. Therefore, for interview methods such as the MMIs we should take into consideration who we select to conduct our interviews; how we train our interviewers; and how we set up scoring criteria for the desired characteristics. In order to reduce the potential for bias based on these demographic differences in opinion, admissions committees should try to include faculty, practitioners, and students of diverse age groups and both sexes in the interview process. The work outlined in this study was designed to determine which personal characteristics should be selected for during our multiple mini-interview process for entry to the DVM Program at OVC. Given the results of this study, when designing our MMI stations, particular attention needs to be paid to the development of the scoring rubrics for each attribute. We cannot create a scenario meant to assess ethical orientation and then only ask the interviewers to just score, “Do you think that this candidate will make a good veterinarian?” It is necessary to provide very clear scoring rubrics that align with the characteristic being assessed. Indeed, recent reports show that well-constructed scoring rubrics increase reliability and inter-rater agreement [8,24,27].

There are limitations to this study. First, there are sample size issues with a 27.86% response rate. However, given the breadth of responses, these values are acceptable. Second, the relative weights and the between-group analyses are generated by the list of eleven characteristics selected by the OVC admissions committee. Had we introduced different or more characteristics, the relative weights may have changed or there may have been no significant differences between various groups on various attributes. However, given the work that the admissions committee did to identify the final list of eleven personal characteristics we feel that the results presented represent attributes that we, and others, believe are important at the time of admission [9].

## Conclusions

This work presents empirical evidence to guide the selection of attributes assessed at the time of admission. While the rank order was relatively consistent between groups, the within characteristic analyses demonstrated that certain attributes are scored higher by certain demographic groups. While the effect sizes are small to medium these findings illustrate how assessors rate applicants may be biased regardless of rater training. In order to reduce the potential for bias based upon these demographic differences in opinion, admission committees must try to include members from all the respective groups (if possible) and provide clear assessment guidelines meant to evaluate the personal characteristics considered important.

## Competing interests

The authors declare that they have no competing interests.

## Authors’ contributions

PC contributed to the design of the study, the write up, and the review and revision of the paper. KH contributed to the design of the study, data analysis, the write up, and the review and revision of the paper. SS contributed to the design of the study and review and revision of the paper. All authors approved the final manuscript.

## Pre-publication history

The pre-publication history for this paper can be accessed here:

http://www.biomedcentral.com/1472-6920/12/105/prepub
